# Study on the differences in aroma components and formation mechanisms of “Nasmi” melon from different production areas

**DOI:** 10.1002/fsn3.2958

**Published:** 2022-07-07

**Authors:** Xupeng Shao, Weizhong He, Yingying Fan, Qi Shen, Jiancai Mao, Meihua Li, Guozhi Hu, Fengjuan Liu, Cheng Wang

**Affiliations:** ^1^ Key Laboratory of Agro‐Products Quality and Safety of Xinjiang, Laboratory of Quality and Safety Risk Assessment for Agro‐Products (Urumqi), Institute of Quality Standards & Testing Technology for Agro‐Products, Xinjiang Academy of Agricultural Sciences Ministry of Agriculture and Rural Affairs Urumqi China; ^2^ College of Food Science and Pharmacy Xinjiang Agricultural University Urumqi China; ^3^ Hami Melon Research Center Xinjiang Academy of Agricultural Sciences Urumqi China; ^4^ Xinjiang Academy of Agricultural Sciences Urumqi China

**Keywords:** aroma, gene, melon, transcriptomics

## Abstract

Aroma is an important factor that guides consumers in purchasing and is thus very important in melon research. To our knowledge, the number of studies with a focus on the aroma differences of the same melon variety in different production areas is largely limited. In this study, the differences in aroma components of “Nasmi” melons from two different production regions were analyzed using gas‐phase ion migration spectroscopy. Transcriptome sequencing was performed for analyzing fragrance‐related genes. Results showed that there were significant differences in the aroma components between products from the two regions. The total amount of aroma compounds from the Turpan region (TT) was 1.7 times higher than that from the Altay region (AT). Through the analysis of transcriptome data, the key genes encoding melon aroma components in different regions were identified as ethanol dehydrogenase, 3‐hydroxyl‐coenzyme A (CoA) dehydrogenase, acyl‐CoA oxidase, long‐chain acyl‐CoA synthetase, acetaldehyde dehydrogenase, and acetyl‐CoA acyltransferase. Real‐time quantitative polymerase chain reaction (RT‐qPCR) showed that the verified genes were similar to the transcriptome. In this study, the main aroma components of the same variety of melon that differed in different production areas and the key genes causing these differences were identified. In addition, the aroma metabolic pathway of melon in different regions was preliminarily elucidated. These results could provide a theoretical basis for further study of the formation mechanism of melon aroma and breeding.

## Graphical abstract

1. aroma synthesis mechanisms of the same melon in different production areas by gas phase ion migration spectroscopy and transcriptome sequencing technology; 2. a total of 29 kinds of aroma components were identified 3. the key genes responsible for the differences in melon aroma components in different regions included ethanol dehydrogenase, 3‐hydroxyl‐CoA dehydrogenase, acyl‐CoA oxidase, long‐chain acyl‐CoA synthase, acetaldehyde dehydrogenase, and acetyl‐CoA acyltransferase; 4. Eleven differentially expressed genes were verified by RT‐PCR, and the results were consistent with the transcriptome data.

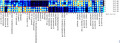

## 1 INTRODUCTION

Melon (*Cucumis melo* L.) is a gourd family (Cucurbitaceae) cucumber (Cucumic) annual trailing herb and is grown on five continents worldwide. According to the FAOSTAT (Food and Agriculture Organization Statistical Database) published by the Food and Agriculture Organization (FAO) of the United Nations, the world's total melon production in 2019 was 27.5013 million tons. China's melon production was 13.5414 million tons, accounting for 49.24% of the world's total production. Xinjiang is one of the main production areas in China, with the melon production ranking first in China year‐round (Xiong et al., 2018). Melon fruit is favored by consumers because of its juicy pulp and unique aroma. Flavor (aroma and taste), color, texture, and nutrients are the main quality determining factors of melon, among which the flavor and color play a leading role in melon consumption (Obando‐Ulloa, Jowkar, et al., 2009). Therefore, study on fruit aroma is very important in melon breeding and quality control. It has been receiving increasing attention from breeders and researchers. The main aroma compounds of melon and their metabolic pathways have been reported by other research groups (Shi et al., 2020).

It has been reported that the aroma of melon fruit is related to development period, variety, postharvest storage, and production area. Beaulieu et al. determined the volatile components of cantaloupe at different developmental stages using headspace solid‐phase microextraction‐–gas chromatography–mass spectrometer (HS–SPME–GC–MS) technology (Beaulieu & Grimm, 2001). They found that the aroma of cantaloupe varied greatly during different developmental stages, and most ester compounds gradually increased with increasing maturity. Lamikanra et al. found that the most prominent volatile compounds in melon were methyl‐butyl acetate and hexyl acetate when stored at 4°C. These two compounds, which contribute to the fruit aroma characteristics of many fruits, are usually present in relatively large proportions in Hami melon (Lamikanra & Richard, 2002). Elazar Falik et al. measured the aroma of two different varieties of melon in Israel and found that C8 fruit had higher levels of aroma volatiles than 5080 fruit (Fallik et al., 2001). Xiao Z et al. determined and analyzed the volatile components of the melon varieties such as Jiashigua, Xizhou Mi 17, and Minqin from different production regions. They detected 45, 46, and 69 volatile compounds in the three cultivars, respectively. Among these volatile compounds, (Z)‐6‐nominal (Z)‐6‐nonenal‐1‐ol, 2‐methyl‐butyl acetate, 3‐methyl‐butyraldehyde, hexanal, and methyl thioacetate were particularly important in Minqin melon (Xiao et al., 2021). While in the study of Hasbullah et al., 3‐pentene‐2‐alcohol, hexyl acetate, and 3‐hydroxy‐2‐butanone were determined to be the key aroma components of Gama Melon Parfum (GMP) melon (Hasbullah & Daryono, 2019).

The main identified metabolic pathways of melon aroma include the fatty acid pathway, amino acid pathway, secondary metabolic pathway, and conversion of alcohols and aldehydes into esters (Lewinsohn et al., 2001; Schwab et al., 2008; Tang et al., 2015). In the fatty acid pathway, straight‐chain aliphatic alcohols, aldehydes, ketones, and esters can be synthesized. Saturated fatty acids are catalyzed by β‐oxidation and acyl‐CoA oxidase to produce lactones. In addition, some unsaturated fatty acids are directly oxidized to form C6 aldehydes and corresponding alcohols and esters by lipoxygenase (LOX). In the amino acid pathway, branched‐chain alcohols and esters are formed mainly through the action of transaminase and dehydrogenase. Aldehydes produced in the above two pathways generate alcohols under the action of alcohol dehydrogenase (ADH). In combination with acyl‐CoA, corresponding esters can be formed under the action of alcohol acyltransferase (AAT). In secondary metabolic pathways, synthesis of some melon volatile phenols and terpenoid substances via the shikimic acid pathway is one of the most important branches. With the combination of these various aroma metabolism pathways, melon forms a unique and strong aroma that appealing consumers love. Related studies have found that enzymes related to melon aroma metabolism mainly include lipoxygenase (LOX), alcohol dehydrogenase (ADH), alcohol acyltransferase (AAT) acyl‐coenzyme A oxidase, aldehyde dehydrogenase, and others (Buchhaupt et al., 2012; Gur et al., 2016; Li et al., 2016a; Shalit et al., 2001).

With rich aroma compound content, “Nasmi” melon which was selected and bred by Academician of Mingzhu Wu was used as the model fruit in our study. Gas chromatography‐ion mobility spectrometry (GC‐IMS) technology was used to determine and analyze the aroma components of melons in different production areas. RNA‐sequencing (RNA‐seq) technology was used to carry out high‐throughput sequencing on fruits of the same “Nasmi” melon from different production areas in Xinjiang. Based on the sequencing data, the genes related to melon fruit aroma metabolism were identified. The formation mechanisms of melon aroma were also subsequently analyzed. These studies may contribute to more fundamental research in melon aroma and industrial practices.

## 2 MATERIALS AND METHODS

### 2.1 Materials

The sampling area is shown in Table 1. The melons with same planting patterns from Turpan are provided by the Xinjiang and Altay experimental base. The same batches of seeds were provided by the Xinjiang Academy of Agricultural Sciences Research Center. Melons were sampled 43 days after pollination. Thirty melons with moderate maturity and no disease/insect pests were selected in the two test regions respectively. Samples were stored at −80°C until use. Three biological replicates were obtained for each sample. The test sample “Nasmi” was planted in two experimental bases, with similar treating and environmental conditions, such as seed pretreatment, plant spacing, seedling stage management, fertilizer, and watering.

**TABLE 1:** Sampling area and number of ‘Nasmi’ melons

The serial number	Sample number	Sampling area	Sampling time	Longitude	Latitude	Instruction
1	AT	Burjin County, Altay, Xinjiang, China	August 26, 2019	89.198021	42.941948	Same variety, different producing area
2	TT	Turpan, Xinjiang, China	May 20, 2019	86.86187	47.704739	

### 2.2 Chemicals and instruments

Polysaccharide polyphenol total RNA extraction kit, FastKing RT Kit (with GDNase), and SuperReal PreMix Plus (SYBR Green) Kit were purchased from Tiagen Biochemical Technology (Beijing) Co Ltd. Diethylpyrocarbonate (DEPC) water, 1,3‐diethyltriazene (DET) buffer (5×), anhydrous ethanol, β‐mercaptoethanol, and anhydrous ethanol were purchased from Shanghai Source Leaf Biotechnology. Primer synthesis is from shanghai BioLeaf Biotech Co Ltd.

The following instruments and equipment were used in this study: medical cryopreservation box (Haier): DW‐86 W100, Haier special electrical appliances, analytical balance (Model No. XSE204), Mettler Toledo (Switzerland), DNBSeq Sequencing Platform (BDA), FlavourSpec® Flavor analyzer (G.A.S. Company, Germany), Agilent 2100 Bioanalyzer (Agilent Technologies, USA), PCR Amplifier (SureCycler 8800, Agilent), and fluorescent PCR instrument (LightCycler 96 Roche).

### 2.3 Method

#### 2.3.1 Melon aroma composition determination

Volatile components were determined by the GC‐IMS technology (Wang, Chen, & Sun, 2019). An automatic headspace (HS) device was used to extract samples. Three‐gram sample was placed in a 20 ml headempty bottle with a magnetic cap, followed by an incubation at 50°C for 15 min. The rotating speed was set to 500 rpm (revolutions per minute). Three hundred microliter sample was injected each time with the injection needle temperature of 55°C. The GC was equipped with a FS‐SE‐54‐CB‐1 capillary column (15 m × 0.53 mm ID, 1 μm). The column temperature stayed at 60°C during the process. The running time was set to 30 min. Nitrogen (99.99%) was used as a carrier gas and its flow rate was initially set at 2 ml/min for 2 min, then increased to 100 ml /min within 18 min and held for 10 min. The 9.8 cm drift tube was operated at constant temperature (45°C) and voltage (5 kV). The flow rate of the drift gas (nitrogen) was set to 150 ml/min. Volatile compounds were identified by comparing the retention index (RI) and the drift time (DT) (the time it takes for ions to reach the collector through drift tube, in milliseconds) of standard in the GC‐IMS library.

#### 2.3.2 Transcriptome sequencing

##### Total RNA extraction from melon fruit

Total RNA extraction was carried out according to the kit operation manual. After extraction, the RNA integrity was detected by an Agilent 2100 biological analyzer.

##### Library preparation and sequencing

Following magnetic bead enrichment of messenger RNA mRNA with Oligo(dT) poly (A), RNA was obtained in segmentation buffer using random N6 primers for reverse transcription, according to the instructions for the retrovirus kit. After that, second strand complementary DNA (cDNA) was synthesized to form double‐stranded DNA. The double‐stranded DNA underwent phosphorylation at the 5′ end and 3′ end to form the sticky end of an “A”. The 3′ end had a bulge in the “T” drum bubble joint, which facilitated connection of the product through PCR amplification with specific primers into a single PCR product following thermal denaturation. Then bridge‐type primers facilitated cyclization of single‐stranded circular DNA to create a single‐strand DNA library. Finally, the DNBSEQ platform was used for sequencing.

#### 2.3.3 qRT‐PCR verification

DNA extraction and reverse transcription were performed according to the instructions of kit. The reaction system was prepared using cDNA as template according to the SuperReal PreMix Plus (SYBR Green) kit. As shown in Table 2, the system volume was 10 μl. Each gene was prepared and analyzed in triplicate. The target gene and internal reference gene were placed in the same plate and measured simultaneously. According to the PCR amplification reaction and melting temperature (Tm) values provided by internal reference genes and target genes, the real‐time fluorescence PCR conditions were set as follows: predenaturation at 94°C for 5 min followed by a cycle with 10S denaturation at 94°C, 30S annealing at 72°C, 15 S extensions, 40 cycles, and 57–95°C melting curve plotted with a heating rate of 1°C/min. The obtained detection results were processed by the 2^−ΔΔCT^ method (Livak & Schmittgen, 2001). The relative contents of multiple differential genes in the same melon fruit from different production areas were calculated to analyze their differential expression. Primer information for 11 different genes designed using Primer 5.0 software is shown in Table 3.

**TABLE 2:** RT‐PCR system

Reagent	Amount
2 × SuperReal PreMix Plus	5 μl
Upstream and downstream primers	0.3 μl each
cDNA	1 μl
RNase‐Free ddH2O	Fill up to 10 μl

**TABLE 3:** Primer design and synthesis

Gene number	Upstream (from 5′–3′)	Downstream (from 5′–3′)
LOC103482546	TCCGAAGAGGCACCCA	GAATCTCCGCCGCAAAC
LOC103483889	TTCCGATCCGAACACTA	TTCCCAATGATGAGACAA
LOC103494755	GCGAAGGACAGGTGCC	ATAAAGGAGCCAAGAGG
LOC103498289	TCGTGAACGGTGGGAGA	CGGATGATCGTCGGAGC
LOC103487507	ACTCGTCAGGTCGTCCAA	ATCAGGCAACTGCGTAT
LOC103494990	TTTTCGGTGGTCAATCT	TCTACAACGAGCAGCAT
LOC103499882	GACGAGGGTATTCGCTTTA	AACACTGGCACGGCTTT
LOC103492479	GGTAGATAAGGCTGGGACA	TCGTAATGGAATGGCTC
LOC107990549	CGTGCCAGATCCATCCC	CCATAAGACTTTAACCAG
LOC103483849	GTTTATCAGGACCTCGTT	TAGCACCAATATCCACATC
LOC103500074	GAGGAGTTGACCGAAGC	AGTTGCCGAAGAATGTA
18SrRNA −1	AGCAAGCCTACGCTCTGT	CTGGTCGGCATCGTTTAT

### 2.4 Data processing

#### 2.4.1 Aroma components

The instrumental analysis software includes LAV (Laboratory Analytical Viewer), three plugins, and GC‐IMS Library Search tools, which can be used for sample analysis from different angles.

#### 2.4.2 Transcriptome data analysis

##### Sequence quality control and cleaning

SOAPnuke V1.5.2 software (https://github.com/BGI‐flexlab/SOAPnuke) was used for filtering and statistical processing of the raw data. The steps were as follows: (I) Removal of reads containing joints (joint contamination); (II) removal of reads with unknown base N content greater than 5%; (III) removal of low‐quality reads. Clean reads were obtained and stored in FASTQ format for subsequent analysis.

##### Quantitative analysis and screening of differential gene expression

Gene expression level, also known as expression abundance, is the first and most important factor in transcriptome data analysis (Sonali et al., 2020). FPKM (fragments per kilobase of transcript per million fragments mapped) was used as an indicator to measure the expression levels of transcripts or genes. The calculation formula is as follows:

$$\text{FPKM}=\left(\text{cDNA Fragments}\right)/\left(\text{Mapped Fragments}\;\left(\text{Millions}\right)\times \text{Transcript Length}\;\left(\mathrm{kb}\right)\right).$$

In this formula, cDNA fragments represent the number of double‐ended reads. Mapped fragments (millions) represent the total number of fragments compared to the transcript. The RNA‐Seq Expectation‐Maximization (RSEM) software package was used to analyze the differentially expressed genes, with different multiples (fold change, FC) of gene expression variation: FC = FPKM (GA)/FPKM (CK). If Log_2_ FC > 0, it was considered to be upregulated. Otherwise, it was considered to be downregulated. The conditions for screening differential genes were that the differential multiple was more than 2 and the corrected *p*‐value was less than or equal to .05 (Love et al., 2014).

##### Functional classification and annotation of differential genes

Gene Ontology and Kyoto Encyclopedia of Genes and Genomes (GO and KEGG) annotation databases are by far the most widely used public databases. The phyper function in R software was used for enrichment analysis of differential genes. The GO classification annotation of differential genes enables us to understand the classification of biological functions of differential genes. The GO database (http://www.geneontology.org/) notes mainly include biological processes, components of cells, and molecular functions. The KEGG database (https://www.kegg.jp/) system includes the functional systematic analysis of intracellular metabolic pathways and gene products, which is helpful for the study of complex gene biological behavior. Analyses on the obtained differential genes based on the KEGG database facilitate the understanding of major metabolic pathways which are demonstrating high gene expression.

## 3 RESULTS

### 3.1 Comparison of aroma components in different production areas

**FIGURE 1:**
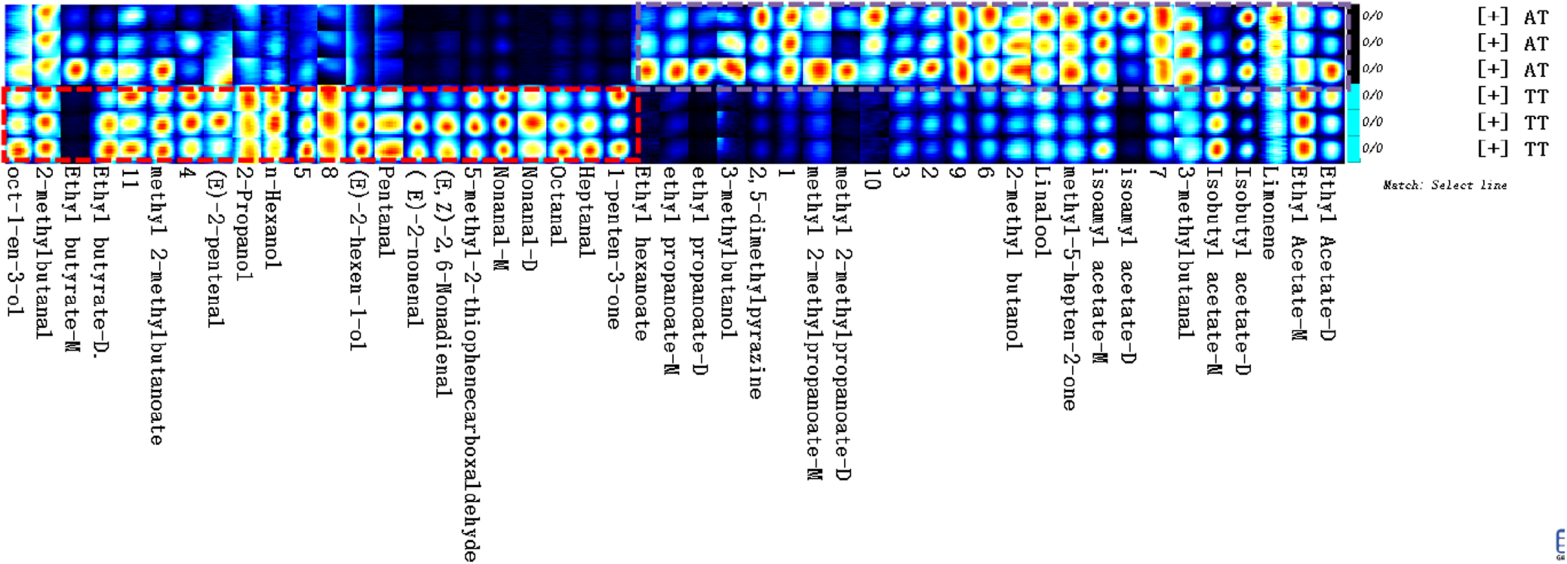
Sample Gallery Plot fingerprint spectrum

Figure 1 and Table 4 show the qualitative and quantitative results of volatile chemicals in melon samples from various production regions. Forty‐seven signal peaks were detected by GC‐IMS, from which 36 typical compounds were identified. The other 11 compounds got no qualitative results due to the limited data in the library database (Table 4). Based on the identified compounds, the volatile compounds in melon samples were esters (8), alcohols (7), aldehydes (10), ketones (2), pyrazines (1), terpenoids (1), and others. As shown in Figure 1, one data point represents one volatile component. However, the same substance could detect monomers or dimers as a result of which one single compound might produce multiple signals. Each row in the figure represents all signal peaks selected from one sample. Each column represents signal peaks of the same volatile organic compounds in different samples. The depth of the color represents the content of the aroma component. The darker the color, the higher the content. The fingerprint contains all signals that can be detected by the instruments. Known substances were marked with existing names, while unknown substances were marked with numbers.

**TABLE 4:** Relative peak areas of 36 volatile components

Classification	Compound	Formula	RI	RT	DT	AT	TT
Aldehyde	(E)‐2‐Nonenal	C_9_H_16_O	1190.2	617.803	1.4095	194.63 ± 12.94	2240.13 ± 89.00
	(E,Z)‐2,6‐Nonadienal	C_9_H_14_O	1177.3	599.417	1.37441	135.77 ± 4.61	883.23 ± 39.82
	5‐Methyl‐2‐thiophenecarboxaldehyde	C_6_H_6_OS	1106.2	498.293	1.17058	236.25 ± 11.49	1776.68 ± 166.96
	Nonanal‐M	C_9_H_18_O	1110	503.701	1.48134	415.67 ± 44.04	2574.36 ± 152.94
	Nonanal‐D	C_9_H_18_O	1109.3	502.62	1.94748	26.20 ± 2.589	310.75 ± 19.39
	Octanal	C_8_H_16_O	1005.2	354.534	1.41052	36.98 ± 3.64	206.98 ± 3.82
	Heptanal	C_7_H_14_O	900.3	262.182	1.32803	40.62 ± 5.11	266.40 ± 5.17
	(E)‐2‐Pentenal	C_5_H_8_O	748.4	182.953	1.35872	25.37 ± 13.87	33.84 ± 1.23
	2‐Methylbutanal	C_5_H_10_O	661.3	151.368	1.16032	337.70 ± 5.17	406.64 ± 13.61
	3‐Methylbutanal	C_5_H_10_O	650.4	148.244	1.17345	140.28 ± 5.87	120.15 ± 5.49
	Pentanal	C_5_H_10_O	695.5	161.955	1.18408	18.37 ± 3.98	52.43 ± 6.71
Esters	Isoamyl acetate‐M	C_7_H_14_O_2_	876.7	247.083	1.2946	184.29 ± 5.18	135.77 ± 13.13
	Isoamyl acetate‐D	C_7_H_14_O_2_	874.6	245.919	1.73557	192.34 ± 12.76	29.21 ± 1.90
	Ethyl butyrate‐M	C_6_H_12_O_2_	795.4	203.2	1.20684	110.61 ± 7.28	20.22 ± 3.98
	Ethyl butyrate‐D	C_6_H_12_O_2_	794.4	202.627	1.56161	346.41 ± 32.38	607.99 ± 19.56
	Isobutyl acetate‐M	C_6_H_12_O_2_	768.1	190.756	1.23076	194.63 ± 13.032	414.81 ± 57.30
	Isobutyl acetate‐D	C_6_H_12_O_2_	766.8	190.248	1.61527	1688.67 ± 114.23	941.45 ± 61.17
	Methyl 2‐methylbutanoate	C_6_H_12_O_2_	773	192.708	1.19313	27.29 ± 16.78	44.22 ± 8.47
	Ethyl acetate‐M	C_4_H_8_O_2_	613.3	137.589	1.09295	323.49 ± 34.47	416.02 ± 6.11
	Ethyl acetate‐D	C_4_H_8_O_2_	611.9	137.202	1.33695	1864.66 ± 110.81	1606.31 ± 130.81
	Methyl 2‐methylpropanoate‐D	C_5_H_10_O_2_	683.3	157.686	1.43755	78.54 ± 9.01	12.86 ± 2.54
	Methyl 2‐Methylpropanoate‐M	C_5_H_10_O_2_	685.7	158.355	1.14213	55.439 ± 2.69	31.38 ± 2.35
	Ethyl propanoate‐M	C_5_H_10_O_2_	706.3	166.213	1.14737	141.81 ± 55.85	47.56 ± 3.61
	Ethyl propanoate‐D	C_5_H_10_O_2_	705.7	165.976	1.45233	216.89 ± 22.63	18.50 ± 3.68
	Ethyl hexanoate	C_8_H_16_O_2_	1003	351.47	1.34079	75.50 ± 3.73	14.60 ± 1.27
Alcohols	2‐Methyl‐butanol	C_5_H_12_O	734	177.226	1.22932	60.46 ± 4.66	40.75 ± 4.64
	3‐Methyl‐butanol	C_5_H_12_O	729.7	175.517	1.48129	125.47 ± 13.93	32.59 ± 2.99
	(E)‐2‐Hexen‐1‐ol	C_6_H_12_O	849.3	232.289	1.18021	79.88 ± 15.43	174.89 ± 13.35
	Oct‐1‐en‐3‐ol	C_8_H_16_O	983.7	331.521	1.16044	43.55 ± 4.478	49.82 ± 7.89
	Linalool	C_10_H_18_O	1092.3	478.403	1.21868	157.20 ± 13.79	117.77 ± 10.32
	n‐Hexanol	C_6_H_14_O	870.2	243.539	1.32576	22.91 ± 1.832	55.04 ± 4.34
	2‐Propanol	C_3_H_8_O	498.5	104.649	1.0888	85.180 ± 11.59	153.62 ± 14.36
Ketones	1‐Penten‐3‐one	C_5_H_8_O	699.3	163.442	1.0779	40.68 ± 3.93	276.97 ± 33.06
	Methyl‐5‐hepten‐2‐one	C_8_H_14_O	992	338.463	1.17649	127.38 ± 6.343	68.57 ± 3.49
Pyrazine	2,5‐Dimethylpyrazine	C_6_H_8_N_2_	875.3	246.307	1.50442	508.60 ± 23.23	114.57 ± 17.23
Terpenes	Limonene	C_10_H_16_	1023.2	380.223	1.21845	110.87 ± 10.60	104.09 ± 5.42

*Note:* M refers to monomer and D refers to dimer.

Abbreviations: DT, the drift time; RI, the retention index; RT, the retention time.

As shown in Figure 1 and Table 4, the eight esters identified in different regions were isoamyl acetate, ethyl butyrate, isobutyl acetate, methyl 2‐methylbutanoate, ethyl acetate, methyl 2‐methylpropanoate, ethyl propanoate, and ethyl hexanoate. As shown in the purple box on the upper right of the map, the concentration of most ester substances in the AT sample was higher than that in the TT sample. Monomers and dimers are recognized in most ester products. According to the peak area, ethyl acetate was the most abundant ester compound in the samples from both production regions. There is no significant difference in ethyl acetate content between TT samples and AT samples. The contents of isobutyl acetate and ethyl propanoate in the AT sample were significantly higher than those in the TT sample. There are 7 alcohols, oct‐1‐en‐3‐ol, linalool, 3‐methyl‐butanol, (E)‐2‐hexen‐1‐ol, 2‐methyl‐butanol, n‐hexanol, and 2‐propanol, which can be detected in this study. The contents of n‐hexanol, (E)‐2‐hexen‐1‐ol, and 2‐propanol in the TT sample were significantly higher than those in the AT samples, while the content of linalool and 3‐methylbutanol in the AT samples was significantly higher than that in the TT samples. Ten aldehyde compounds, (E)‐2‐nonenal, (E, Z)‐2,6‐nonadienal, 5‐methyl‐2‐thiophenecarboxaldehyde, nonanal, octanal, heptanal, (E)‐2‐pentenal, 2‐methylbutanal, 3‐methylbutanal, and pentanal, were detected. As shown in the red box, the content of most aldehydes in the TT sample was higher than that in the AT sample. The 3‐methylbutanal is the only aldehyde which showed a slightly higher content in AT samples than in TT samples. The content of (E)‐2‐nonenal in TT was 11.5 times higher than that in the AT sample. The contents of nonanal, octanal, (E, Z)‐2, 6‐nonadienal, and heptanal in the TT sample were significantly higher than those in the AT sample. Additionally, two different types of ketones, methyl‐5‐hepten‐2‐one and 1‐penten‐3‐one, were detected by our instruments. The content of methyl‐5‐hepten‐2‐one was higher in AT samples. The 1‐penten‐3‐one content of TT samples was nearly seven times higher than that in AT samples. The contents of 2,5‐dimethylpyrazine (pyrazine) and limonene (terpene) were higher in AT samples than in TT samples.

### 3.2 Transcript mapping

Sequencing was performed on the TT samples and AT samples. The obtained data are shown in Table 5. More than 1 million original reads were obtained from all the 6 samples. The filtered clean reads ratio was above 99%. The proportion of the number of bases with a quality value greater than 20 in filtered reads was above 97%. More than 91% of the bases have a mass value greater than 30.

**TABLE 5:** Statistical results of melon transcriptome sequencing data in different regions

Sample	Total raw reads (M)	Total clean reads (M)	Total clean bases (Gb)	Clean reads Q20 (%)	Clean reads Q30 (%)	Clean reads ratio (%)
AT_1	105.65	104.73	10.47	98.12	91.67	99.14
AT_2	103.19	102.88	10.29	97.85	91.13	99.7
AT_3	103.19	102.83	10.28	97.86	91.12	99.65
TT_1	103.19	102.77	10.28	98.19	91.85	99.6
TT_2	103.19	102.79	10.28	98	91.69	99.61
TT_3	103.19	102.83	10.28	98.14	91.67	99.65

### 3.3 Differential gene identification

**FIGURE 2:**
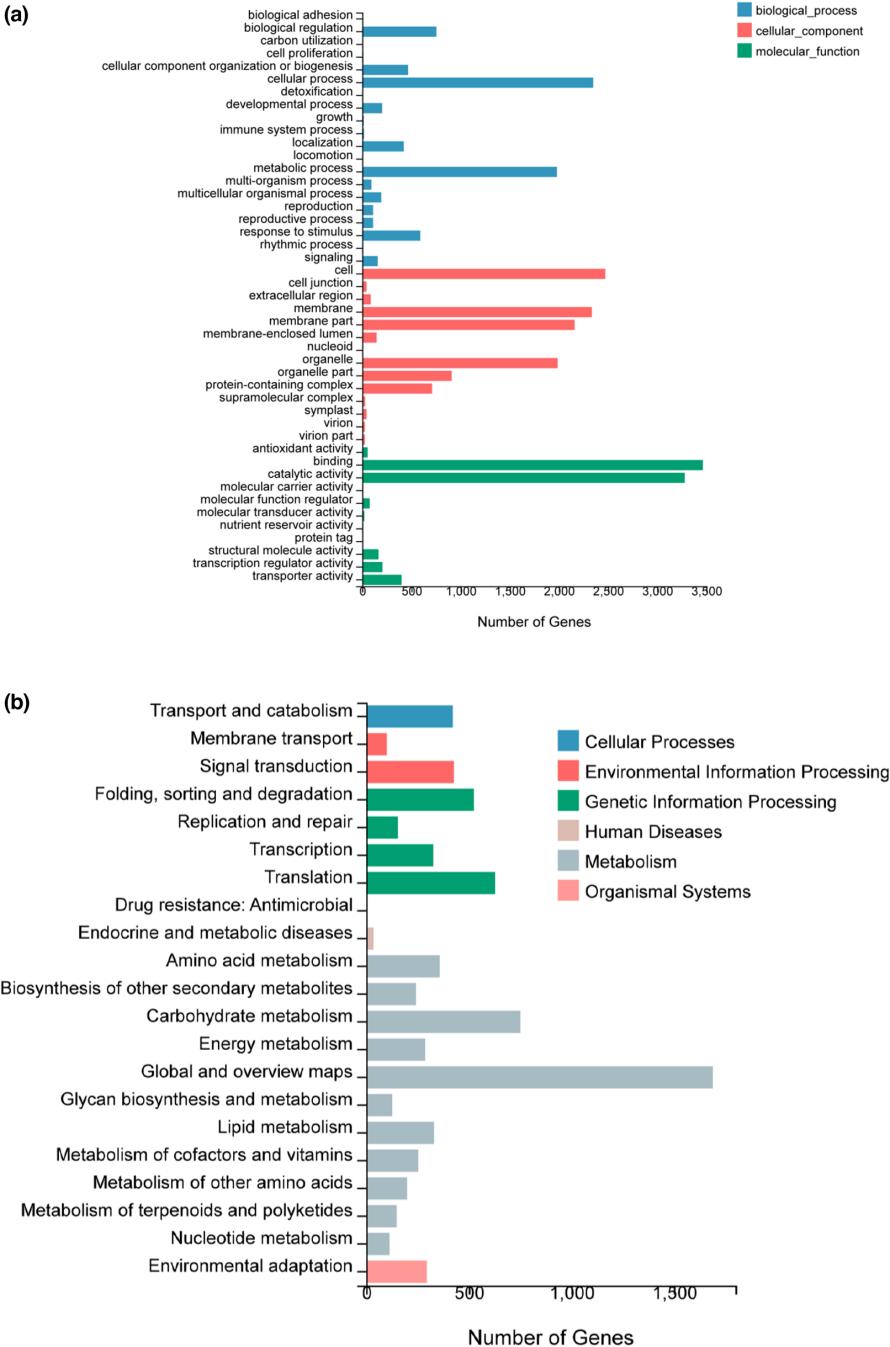
Differential Gene Ontology (GO) (a) and Kyoto Encyclopedia of Genes and Genomes (KEGG) (b) classification

According to differential gene identification, a total of 9358 differentially expressed genes were found in “Nasmi” melon from the two regions, among which 4621 genes were upregulated and 4737 genes were downregulated. The obtained differentially expressed genes were used for subsequent analysis. As shown in Figure 2a, these genes were annotated according to GO functions. In the biological process, genes which play roles in biological regulation, cellular process, metabolic process categories, and the response to stimulus enrichment were relatively high (754, 2349, 1980, and 590, respectively). The least annotated entry was growth‐related genes, among which 8 genes were upregulated and 7 were downregulated. In addition, a total of 15 genes were enriched and annotated in this group of genes. Among the cell component related genes, there were more annotated genes in cell, membrane, membrane part, and organelle, with a number of 2472, 2335, 2160, and 1987, respectively. Virions got fewest gene enrichment notes among cell components, with only 26 differential genes, 10 of which were upregulated. Among molecular function related genes, the differentially expressed genes enriched in binding and catalytic activity annotations were far more than other entries, with values of 3464 and 3280, respectively. Among them, 1470 genes were upregulated in binding, and 1594 genes were upregulated in catalytic activity. As shown in Figure 2b, the obtained differentially expressed genes were classified into KEGG biological pathways. A total of 4061 differentially expressed genes were classified into different groups involved in 21 pathways. These groups of genes are mainly associated with certain biological functions, such as cellular processes, environmental information processing, genetic information processing, metabolism, organizational systems, and human disease. “Metabolism” was the major category for a single gene. Further division of each category showed the biggest differences in the “global and overview map” group (1690), followed by the “carbohydrate metabolism” group (751) and “translate” group (628). Some other groups, such as “fold, classification and degradation,” “transportation and catabolism,” and “signal transduction” gene annotation, are also showing substantial differences.

### 3.4 KEGG annotation pathway enrichment

**FIGURE 3:**
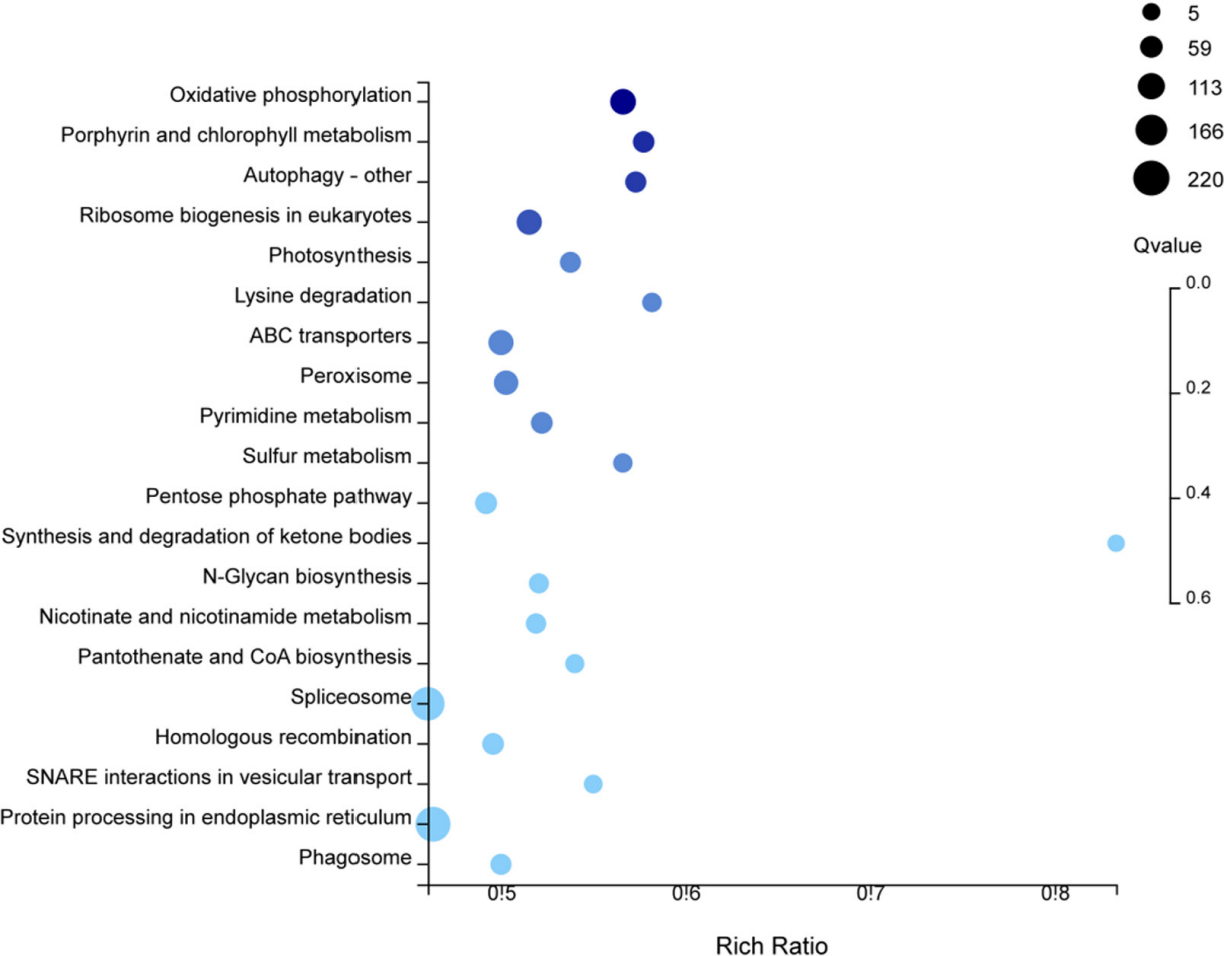
Kyoto Encyclopedia of Genes and Genomes (KEGG) pathway enrichment of different genes in melon in different regions

As shown in Figure 3, Enrichment analysis showed that these differentially expressed genes were enriched in 136 metabolic pathways, including 10 pathways with a *p*‐value ≤.05 and only 1 pathway with a *Q* value ≤0.05. The top 20 pathways with low *Q* values are shown in Figure 3. The oxidative phosphorylation was significantly enriched (*Q* value ≤0.05). There are 107 genes that were enriched to medium level, of which 67 genes were upregulated. The pathways of the most enriched genes were RNA transport and endocytosis, with 248 (95 upregulated) and 244 (108 upregulated) genes, respectively.

### 3.5 Genes related to aroma metabolism of melon from different production areas

Fatty acid metabolism in the melon aroma formation process plays a very important role. Transcriptional omics data showed that a total of five pathways related to the fatty acid metabolism pathway are involved. Pyruvate metabolism and a large amount of amino acid metabolism are also associated with aroma composition. The expression of genes enriched in some of the metabolic pathways related to aroma is shown in Table 6.

**TABLE 6:** Pathways related to fatty acid metabolism

KEGG pathway	Differences in gene expression		
	Up	Down	All
Fatty acid degradation	26	16	42
Fatty acid biosynthesis	11	14	25
Fatty acid elongation	8	13	21
Biosynthesis of unsaturated	8	10	18
Fatty acid metabolism	26	17	43
Pyruvate metabolism	36	48	84

Some representative differential genes are shown in Table 7. Acetyl‐CoA, the hub of various biological metabolism pathways, is also the precursor of the synthesis of aroma components. Its synthesis mainly occurs via oxidative decarboxylation of pyruvate produced by the glycolysis reaction or β‐oxidation of fatty acids. According to the transcriptome analysis based on aroma metabolism, acyl‐CoA ‐ LOC103482592 oxidase genes differentially expressed in the TT samples and AT samples (4.576 and 51.336, respectively). The expression of LOC103483849 and LOC103500074 encoding alcohol dehydrogenase was significantly different between TT samples and AT samples. Of the 7 genes encoding aldehyde dehydrogenase (ADH), 6 were significantly upregulated in samples from the two regions. The differential gene LOC103500505 encoding acetyl‐CoA c‐acetyltransferase was significantly upregulated in TT samples (553.813) while not in AT samples (111.556). The expression level of LOC103487805 encoding branched‐chain amino acid aminotransferase was significantly different between the two groups, with 25 times higher value in AT samples than in TT samples. In addition, a large number of different genes related to the synthesis of coenzyme A were also found, which play an important role in the synthesis of aroma precursors.

**TABLE 7:** Selected genes related to melon fruit aroma climate in different regions

Gene ID	FPKM(TT)	FPKM(at)	Description [EC:NO]
LOC103482592	24.576	51.336	Acyl‐CoA oxidase [EC:1.3.3.6]
LOC103482821	8.193	2.593	Long‐chain acyl‐CoA synthetase [EC:6.2.1.3]
LOC103486958	44.22	52.156	Long‐chain acyl‐CoA synthetase [EC:6.2.1.3]
LOC103487766	867.386	5647.706	Acyl‐[acyl‐carrier‐protein] desaturase [EC:1.14.19.2 1.14.19.11 1.14.19.26]
LOC103483284	146.956	66.893	Acetyl‐CoA carboxylase carboxyl transferase subunit alpha [EC:6.4.1.2 2.1.3.15]
LOC103500505	111.556	553.813	Acetyl‐CoA C‐acetyltransferase [EC:2.3.1.9]
LOC103497788	62.923	187.83	Enoyl‐CoA hydratase [EC:4.2.1.17]
LOC103501532	73.04	5.46	Fatty acyl‐ACP thioesterase B [EC:3.1.2.14 3.1.2.21]
LOC103500074	174.696	2261.113	Alcohol dehydrogenase [EC:1.1.1.1]
LOC103483849	1639.03	5491.8	Aldehyde dehydrogenase (NAD+) [EC:1.2.1.3]
LOC103483275	26.67	41.816	Acyl‐coenzyme A thioesterase 1/2/4 [EC:3.1.2.2]
LOC103496981	61.103	205.602	Acetyl‐CoA acyltransferase 1 [EC:2.3.1.16]
LOC103497860	114.62	206.155	3‐hydroxyacyl‐CoA dehydrogenase [EC:1.1.1.35 1.1.1.211]
LOC103501381	14.82	66.556	Acetate‐CoA ligase [EC:6.2.1.1]
LOC103498837	45.006	11.47	Pyruvate dehydrogenase E1 [EC:1.2.4.1]
LOC103488170	64.613	624.273	Malate dehydrogenase (oxaloacetate‐decarboxylating) (NADP+) [EC:1.1.1.40]
LOC103499065	26.333	44.133	Malate dehydrogenase (decarboxylating) [EC:1.1.1.39]
LOC103487805	39.116	977.476	Branched‐chain amino acid aminotransferase [EC:2.6.1.42]

**FIGURE 4:**
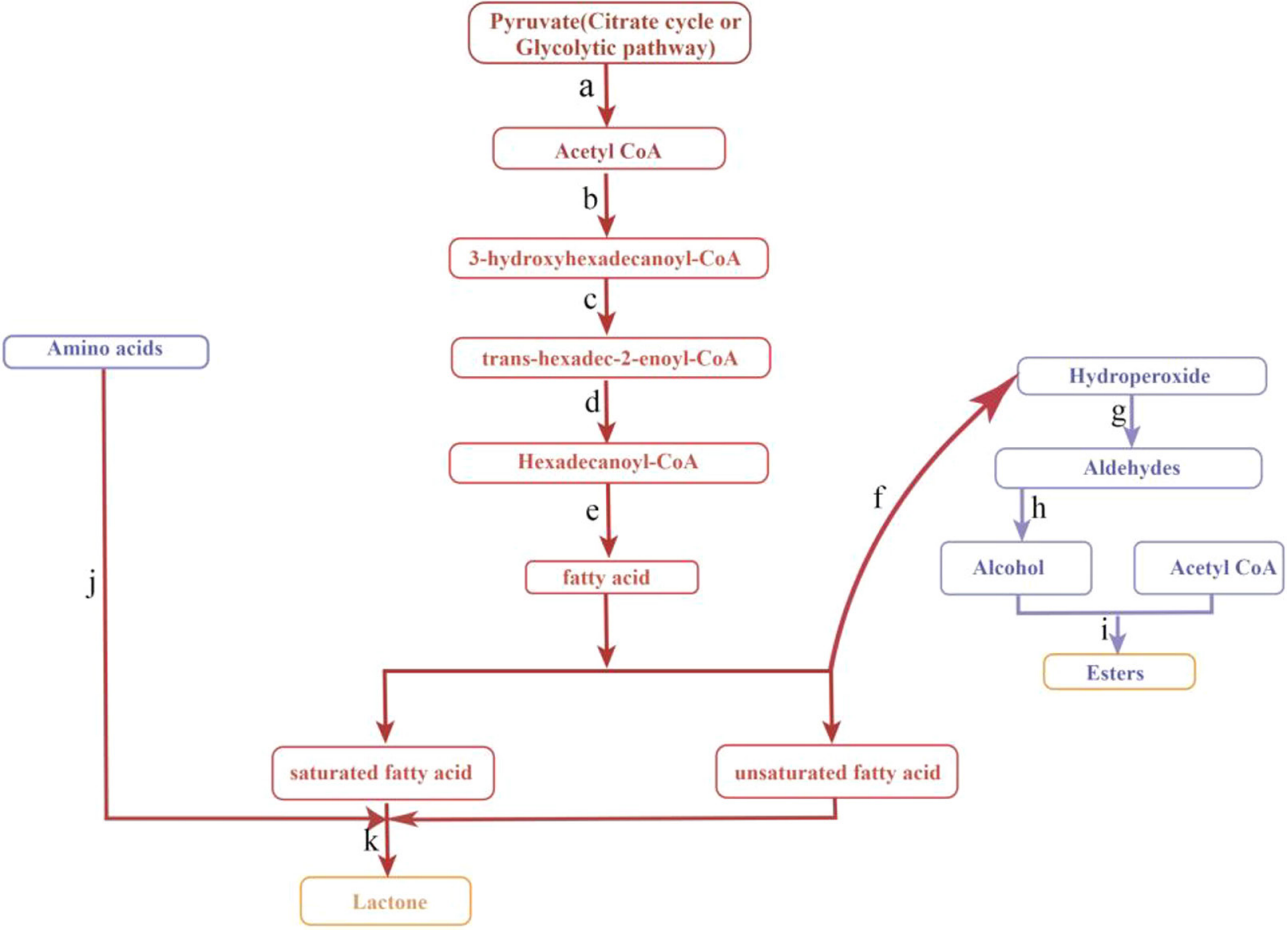
Melon aroma metabolism pathway. The small letters in the figure represent the related enzymes involved in the reaction, and one letter represents one or more enzymes. (a) pyruvate dehydrogenase [EC:1.2.4.1] and aldehyde dehydrogenase [EC:1.2.1.3]; (b) acetyl‐CoA C‐acetyltransferase [EC:2.3.1.9], acetyl‐CoA acyltransferase 1 [EC:2.3.1.16], and 3‐hydroxyacyl‐CoA dehydrogenase [EC:1.1.1.35 1.1.211]; (c) enoyl‐CoA hydratase [EC:4.2.1.17]; (d) acyl‐CoA oxidase [EC:1.3.3.6]; (e) long‐chain acyl‐CoA synthetase [EC:6.2.1.3]; (f) lipoxidase [EC:1.13.11.12]; (g) hydroperoxide lyase [EC:4.1.2.‐]; (h) alcohol dehydrogenase [EC:1.1.1.1]; (i) alcohol acyltransferase [EC:2.3.1.‐]; (j) branched‐chain amino acid aminotransferase [EC:2.6.1.42], 2‐oxoisovalerate dehydrogenase E1 component beta subunit [EC:1.2.4.4] and 2‐oxoisovalerate dehydrogenase E2 component [EC:2.3.168]; (k) acyl‐CoA oxidase [EC:1.3.3.6] and acid hydroxylase [EC:1.14–] fatty acid hydroxylase

As shown in Figure 4, studies on aroma metabolic pathways have preliminarily demonstrated the metabolic pathway of melon fruit aroma (Beaulieu, 2006; Gonda et al., 2010; Lange et al., 2000; Mayobre et al., 2021; Song et al., 2021). The precursor substance pyruvate is synthesized from glycolysis or organic acid metabolism. Under the action of pyruvate decarboxylase (PDC), acetyl‐CoA, which is crucial to aroma metabolism, is formed. Acetyl‐CoA produces 3‐oxohexadecanoyl‐CoA through the action of acetyl‐CoA acyltransferase 1, which is then transformed into 3‐hydroxyhexadecanoyl‐CoA through 3‐hydroxyl‐CoA dehydrogenase. Transhexadec‐2‐enoyl‐CoA is formed by enyl‐CoA hydrase, which generates hexadecanoyl‐CoA under the action of acyl‐CoA oxidase, and fatty acids can be formed under the action of long‐chain acyl‐CoA synthetase. At the same time, fatty acids can be interconverted with aldehydes via aldehyde dehydrogenase (nicotinamide adenine dinucleotide (NAD+)). While aldehydes can be interconverted with alcohols under the action of alcohol dehydrogenase (ADH). Alcohol acyltransferase (AAT) plays an important role in ester synthesis by transferring the acyl group of acetyl‐CoA to alcohols to produce ester compounds. A variety of amino acids are degraded into branched‐chain fatty acids by transaminase and dehydrogenase, resulting in ester compounds.

### 3.6 Real‐time quantitative RT‐PCR verification

To verify the transcriptome data, we randomly selected 11 genes for real‐time fluorescence quantitative verification. As shown in Table 8, annotated information, expression levels obtained from transcriptome data, and relative contents obtained using real‐time fluorescence quantitative technology for 11 verified genes are presented. The results showed that the 11 verified genes were consistent with the transcriptome data, indicating the high reproducibility and reliability of transcriptome analysis.

**TABLE 8:** RT‐PCR verification of melon‐related genes in different regions

Gene ID	Description	FPKM TT	FPKM AT	FPKM (TT/AT)	Relative content of RT‐PCR (TT/AT)
LOC103483889	Fructokinase	127.626	179.14	0.712	0.788 ± 0.025
LOC103482546	6‐phosphofructokinase 1	15.75	38.666	0.407	0.629 ± 0.031
LOC103494755	Sucrose synthase	481.56	286.346	1.682	1.216 ± 0.028
LOC103498289	Alpha‐glucosidase	31.29	12.426	2.518	2.324 ± 0.065
LOC103487507	Malate dehydrogenase	38.293	15.503	2.470	1.896 ± 0.006
LOC103499882	Citrate synthase	97.44	136.803	0.712	0.521 ± 0.027
LOC103494990	Aconitate hydratase	64.893	40.03	1.621	1.233 ± 0.031
LOC103492479	L‐ascorbate oxidase	28.875	251.22	0.115	0.357 ± 0.003
LOC107990549	L‐gulonolactone oxidase	7.026	1.256	5.594	3.255 ± 0.039
LOC103500074	Alcohol dehydrogenase	174.696	2261.113	0.077	0.121 ± 0.012
LOC103483849	Aldehyde dehydrogenase	1639.036	5491.83	0.298	0.512 ± 0.035

## 4 DISCUSSION

### 4.1 Influence of region on melon aroma components

Aroma can be classified into different types based on different proportions of aroma components, such as flower flavor, green flavor, fruit flavor, aldehyde flavor, and others. Flower fragrance refers to substances with flower fragrance. Green flavor refers to the aroma of fresh grass, mainly containing C6 aldehydes, C9 aldehydes, and alcohols. Fruity flavor is mainly represented by ripe apples, strawberries, and other odors, including phenols, ethers, and esters (Selli et al., 2012). Aldehyde flavor mainly manifests as melon odor substances, which are represented by C7 to C12 aliphatic aldehyde substances. In melon, the main characteristic aromas are fruit flavor and green flavor. The results showed that the fruit aroma in different regions was significantly different as a result of different altitudes, temperatures, light conditions, and soil environments. For example, Xiao Z et al. compared and analyzed the volatile components of melon from Jiashigua, Xizhou Mi 17, and Minqin in Xinjiang. They found that there were great differences in the types and contents of aroma components among different producing areas and varieties (Fallik et al., 2001). Zehra Guler measured volatile components of 3 groups of melon samples from different regions of Turkey. The results showed that the volatile components of melon in the three groups were significantly different in both quality and quantity (Güler et al., 2013). Melon is a photophilous and thermophilic species. Different temperatures and light have notable effects on melon production and quality. The optimal temperatures for melon growth and maturation are 27–30°C in the daytime and approximately 18°C at night. The light compensation point of melon is approximately 4000 lx, while the light saturation point is approximately 55,000 lx. Under sufficient light conditions, the melon plant grows robustly. There were significant differences in temperature, illumination, altitude, and other conditions between the products from two regions in this study. This is the main cause of the significant differences in aroma components of the same variety of “Nasmi” melon. Not all aroma ingredients are related to fragrance. Some characteristic aroma contents are very high, but it is difficult for us to smell because of the high flavor threshold of the odor. Those with a high aroma value (the ratio of aroma components to aroma threshold) are called “characteristic effector compounds.” The characteristic aroma compounds of melon have been identified as hexyl acetate, ethyl caproate, ethyl butyrate, ethyl 2‐methyl butyrate, ethyl 2‐methyl propionate, ethyl 3‐methyl butyrate, (Z, Z)3,6‐nonenyl acetate, (Z)‐6‐nonenal, (E, Z)‐2,6‐nonenal, (E)‐2‐nonenal, 2‐(methylethyl) ethyl acetate, 3‐(methylethyl) propionate ethyl ester, and (Z)‐1,5‐octadiene‐3‐ketone (Obando‐Ulloa et al., 2008; Obando‐Ulloa, Nicolai, et al., 2009). Some of these characteristic effector compounds were identified in the melons used in this study.

### 4.2 Aroma metabolic main way

In recent years, research on aroma has attracted great attention from many research groups. The main pathways of fruit aroma synthesis can be divided into the fatty acid pathway, secondary metabolic pathway, amino acid pathway, and conversion of alcohol and aldehyde compounds to esters. In this study, all four pathways were identified. More emphases were put on the fatty acid pathway in our analysis. Amino acid pathways have been found to play an important role in the synthesis of branched‐chain esters, in which valine, leucine, and isoleucine are important precursors. Yan Li et al. analyzed the amino acid pathway during the synthesis of melon aroma and found that the amino acid pathway mainly depended on the decomposition of leucine, isoleucine, valine, phenylalanine, and cysteine into aroma volatile substances through transamination and dehydrogenation (Li et al., 2016b). In the fatty acid pathway, pyruvate is an important precursor substance. Under the action of pyruvate decarboxylase (PDC) and alcohol dehydrogenase (ADH), pyruvate degrades to synthesize acetyl‐coenzyme A, which provides a precursor for the production of fatty acids. Min Min Wang et al. studied PDC1 (pyruvate decarboxylase 1) in melons and found that maturation‐induced PDC1 encodes a pyruvate/α‐ketoate decarboxylase, which is involved in the biosynthesis of acetaldehyde, propionate, and valeraldehyde in melon fruits and plays an important role in the decarboxylation of pyruvate and 2‐oxy‐caproate (Wang, Zhang, et al., 2019). Liu et al. (Liu et al., 2020) resequenced 297 melon materials to reveal the genome improvement history of melon and the loci related to fruit traits. This study found that the Cm AAT gene was a special gene in melon fruits and was closely related to aroma formation. CM AATS plays an important role in the last step of ester biosynthesis, leading to the synthesis of various ester aromas (Chen et al., 2016). The high content of alcohols and volatile esters in melon is related to the activity of CMAAT (Melo3C024771), which determines the high content of alcohols and volatile esters in melon volatiles (Galaz et al., 2013). This study showed that the action of AAT produces alcohols and acetyl in combination with acetyl coenzyme A, resulting in esters. Additionally, this study found that alcohol and aldehyde compounds result in the transformation of esters. In this way, there is mutual transformation between material, alcohols, and aldehydes, which are subsequently proceeded to be involved in the formation of esters, or fatty acids proceed through aldehyde dehydrogenase and participate in the fatty acid pathway to form esters. The secondary metabolic pathways of aroma metabolism can be divided into terpene pathways and the synthesis of volatile phenols. In terpene pathways, the main enzyme is terpene synthase, under which semiterpenes, polyterpenes, and other terpenes are synthesized (Portnoy et al., 2008). In addition, shikimic acid can synthesize many aromatic compounds, such as coumarin and flavonoids, through the shikimic acid pathway. Song and Forney et al. found that benzyl acetates are one of the major ester groups in melon, and this ester is usually synthesized through the shikimic acid pathway (Song & Forney, 2008). Additionally, many sulfur compounds contribute to melon flavor, but they have not been identified in “Nasmi” melon in this study due to the fact that the specific melon varieties were used, or the incomplete records in the IMS database.

### 4.3 Melon aroma content and amount of gene expression in different regions

Aroma content is regulated by a variety of different genes. In this study, transcriptome technology was used to identify the key genes responsible for the difference in aroma metabolism in different production areas. Aroma components and relative contents between production areas were determined by the GC‐IMS technology. Combined analysis showed that aroma content was inextricably related to these key genes. In aroma metabolism, pyruvate dehydrogenase is an important enzyme in the process of pyruvate conversion to acetyl‐CoA. The expression levels of several differential genes encoding pyruvate in melon from different production areas changed significantly between the two regions. The expression levels of most of them in the TT samples were significantly higher than those in the AT samples. The same aroma metabolic pathways encoding aldehyde dehydrogenase in eight different genes express seven upregulated genes in TT samples compared to AT samples. The total aldehyde material in AT samples is relatively lower than that in TT samples, which may indicate a specific correlation between these observations. Results in alcohol dehydrogenase (ADH) (multiple genes are upregulated in TT vs AT) are in agreement with the observation that the content of aroma ingredients found in quantitative AT relative content is lower than that in TT samples. For the acetyl‐CoA ‐ LOC103496981 acyltransferase gene, the expression in TT and AT samples was 61.103 and 205.602, respectively. This gene is closely related to ester synthesis, which may be the reason why the content of most ester compounds in AT is higher than that in TT. Through gene screening, comparison, and analysis of aroma quantities, the relevant contents of aroma demonstrated a positive correlation between the expression levels of key genes and their effect, thus facilitating preliminarily evaluation. Because of the roles of these key genes, the aroma composition between the two regions shows large differences.

This study focused on the fatty acid and amino acid pathways of melon aroma metabolism. We discovered that the genes primarily involved in aroma metabolism were acetyl‐CoA acyltransferase, 3‐hydroxyl‐CoA dehydrogenase, acyl‐CoA oxidase, long‐chain acyl‐CoA synthetase, aldehyde dehydrogenase, and alcohol dehydrogenase in transcriptome analysis, which is consistent with previous research (Li et al., 2011; Zhang et al., 2017). At the same time, based on several aroma studies and the current study, we preliminarily obtained the synthesis pathways of aroma components, which provides strong support for melon breeding and other related studies.

## 5 CONCLUSION

By studying the volatile components of the same melon from different production areas, it was observed that there was no difference in the types of volatile components of the melon from the two regions. However, there was a notable difference in the aroma contents. The main components that differed between the two regions were esters, alcohols, and aldehydes. The total aroma of the TT sample was 1.7 times higher than that of the AT sample. In the two regions, with the same variety of melon, transcriptome sequencing analysis produced 9658 different genes, and through the classification based on GO annotations, the three major categories all demonstrated notable amounts of enrichment. Through the analysis of aroma‐related pathways and KEGG analysis, a large number of fruit‐related metabolic pathways were obtained. Investigation of aroma and analysis of multiple genes related to regional differences identified the acetyl‐CoA acyltransferase, 3‐hydroxyl acyl‐coenzyme A, dehydrogenase, acyl‐coenzyme A oxidase long‐chain acyl‐coenzyme A synthetase, acetaldehyde dehydrogenase, and alcohol dehydrogenase genes. Some differentially expressed genes were verified by real‐time fluorescence PCR. The results showed that the transcriptome sequencing results were reliable.

## CONFLICT OF INTEREST

The authors declare no conflicts of interest.

## Data Availability

The data that support the findings of this study are available from the corresponding author upon reasonable request.
